# Mercury and selenium in fish of Fountain Creek, Colorado (USA): possible sources and implications

**DOI:** 10.1186/s40064-016-2088-6

**Published:** 2016-04-12

**Authors:** D. R. Nimmo, S. J. Herrmann, J. S. Carsella, C. M. McGarvy, H. P. Foutz, L. M. Herrmann-Hoesing, J. M. Gregorich, J. A. Turner, B. D. Vanden Heuvel

**Affiliations:** Department of Biology, Colorado State University-Pueblo, Pueblo, CO 81001 USA; Department of Chemistry, Colorado State University-Pueblo, Pueblo, CO 81001 USA; Division of Colorado Parks and Wildlife, 6060 Broadway, Denver, CO USA; Department of Veterinary Microbiology and Pathology, Washington State University, Pullman, WA 99164 USA

**Keywords:** Fountain Creek watershed, Fish contamination, Mercury, Selenium, Water quality

## Abstract

Fountain Creek in Colorado USA is a major tributary that confluences with the Arkansas River at Pueblo, Colorado, the result being the tributary’s influence on Arkansas River water quality affecting down-stream users. In a previous study, we found that bryophytes (aquatic plants) accumulated selenium in Fountain Creek watershed and this finding prompted us to investigate the extent of the metalloid in the whole-body tissues of fish. One hundred 11 fish (six species) were collected and analyzed for Se by inductively-coupled plasma emission mass spectrometry. Analysis of all analytical data also showed mercury in all of the fish whole bodies and selected tissues. There was a general increase in selenium but a decrease in mercury in fish with downstream travel-distance. The highest whole-body selenium was in Pueblo, Colorado (3393 µg/kg, dry weight; 906 µg/kg, wet weight); the highest mercury in fish was in the Monument Creek tributary north of Colorado Springs, Colorado (71 µg/kg, dry weight; 19 µg/kg, wet weight). In four tissues of 11 female fish captured, selenium was highest in the livers at eight sites but highest in the ovaries at three sites. Mercury was highest in the epaxial muscle at all sites. Selenium availability could be due to the watershed lithology and land uses; however, mercury could be carried by atmospheric deposition from coal-fired power plants and historic mining activities. Selenium in fish tissues and water samples were compared to U.S. national water quality criteria.

## Introduction

Selenium (Se) has been an element of concern in the Fountain Creek watershed that drains the Pike’s Peak-Colorado Springs area for many years. Fountain Creek drains much of El Paso County, the most populous county in Colorado with over 560,000 residents (Salazar 2006) and growth has resulted in several water-quality issues affecting downstream counties including the City and County of Pueblo, Colorado. Salazar’s (2006) vision statement included high selenium content as a major issue in Fountain Creek along with others such as wastewater spills, non-point pollution, high coliform levels, scrubbed waste water return flows, destructive farm and ranch runoff, loss of wetlands and a host of other issues. Among other issues were flash floods leading to erosion, resulting in questions about the sources of Se, the movement and varying concentrations of Se in the watershed, and was there uptake of the metalloid into the stream biota including fish? Because of an earlier study by Nimmo et al. (2006) who showed the cadmium and zinc accumulation by an aquatic plant species, *Hygrohypnum ochraceum,* in the upper Arkansas River, Colorado, we used *H. ochraceum* as an indicator organism to determine Se-uptake in the Fountain Creek watershed (Herrmann et al. 2012). Four findings of this effort were that the: (1) plants obtained more Se in the basin with travel-distance downstream, (2) plants showed a trend to take up more Se with total hardness in the spring versus the fall, (3) bryophytes accumulated Se thousands of times above the Se-concentration in ambient water, particularly noticeable in the upper reaches of the watershed in the fall, and most important, (4) the bryophytes appeared to be a suitable indicator of Se bioavailability to other trophic levels. As a result, primary questions to be addressed in this study were (1) could Se be detected in fish populations throughout the Fountain Creek watershed and if so, (2) to what extent was the Se being accumulated? Because of the initial findings with the bryophytes, we conducted a reconnaissance of fish populations in the watershed March, April and May of 2009 using inductively-coupled plasma mass spectrometry (ICP-MS) to analyze selenium (if any) in fish tissues. Surprisingly, we detected mercury (Hg) in the fish as well as Se; therefore, in this report we included and discussed the findings and possible implications of mercury in fish.

### Description of study area

**Fig. 1:**
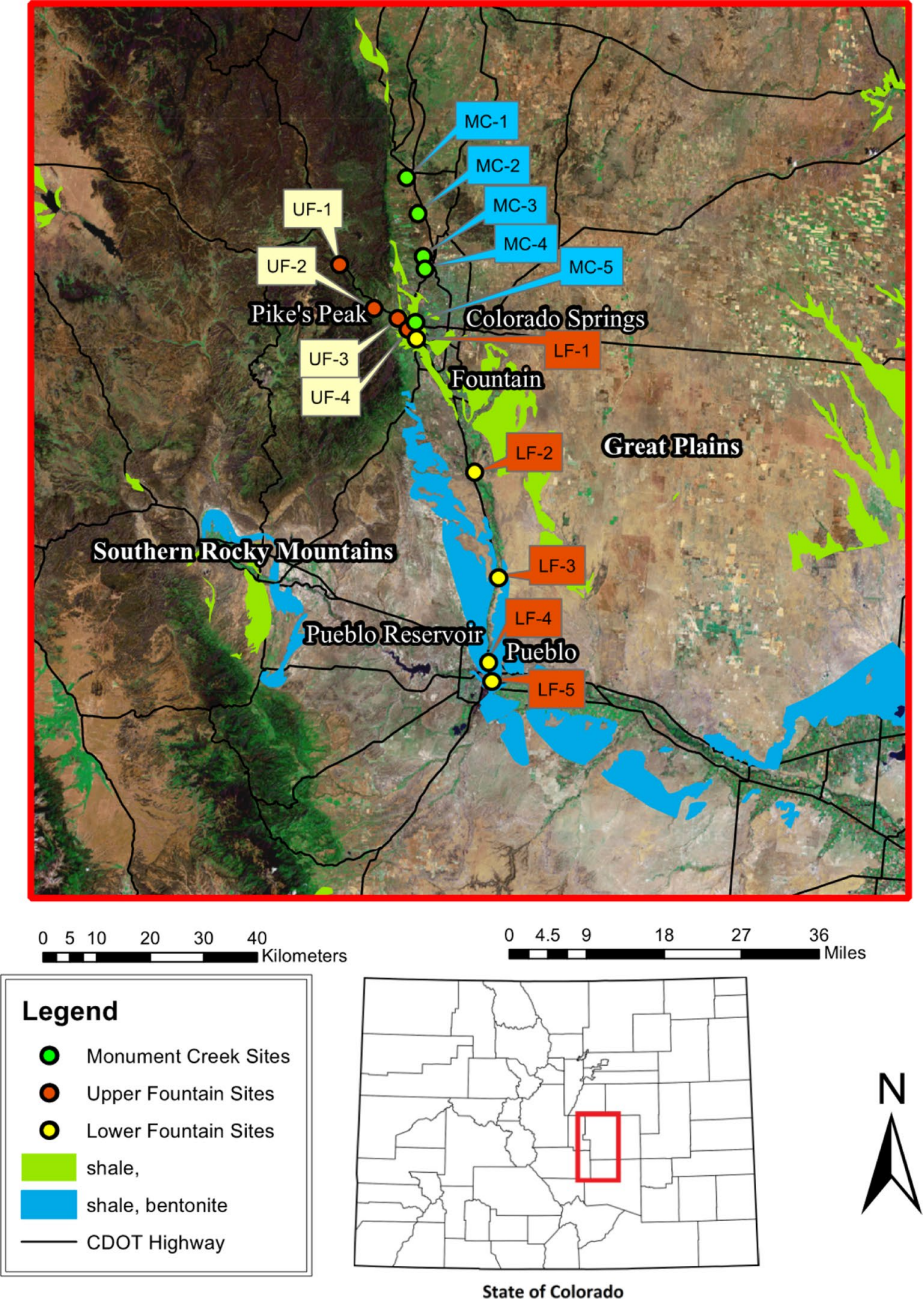
Map of 14 site locations in study area: Fountain Creek watershed, Colorado. The watershed is in the form of a “Y”; therefore the sites in the Upper Fountain Creek segment are shown as white squares; those in the Monument Creek Tributary segment are shown as blue; and, those in the Lower Fountain Creek segment are indicated as *red*. Locations, coordinates and elevations of each study site are listed in Table 1

The Fountain Creek watershed drainage area is 2398 km^2^ (Bruce 2002) bounded by the metropolitan area of Colorado Springs, Colorado on the north and Pueblo, Colorado on the south (Fig. 1). Elevation ranges from 4300 m at the summit of Pikes Peak to 1432 m at the Creek’s confluence with the Arkansas River in Pueblo, Colorado. Upper Fountain Creek flows through sand, gravel, and boulders underlain with sandstone and shale; after leaving the mountains, it becomes a typical meandering sandy-bottom warm-water stream free of any impoundments, dams or reservoirs along its course. Herrmann et al. (2012) found Fountain Creek to be a well-oxygenated stream with median dissolved oxygen concentrations of 14 sites (11.2 mg/l in the spring and 8.7 mg/l in the fall). A major tributary to Fountain Creek, Monument Creek, originates in the Rampart Range of Colorado flowing easterly until it leaves the front range mountains then the tributary turns south eventually confluencing with the upper Fountain Creek at America the Beautiful Park in downtown Colorado Springs, Colorado. The confluence of the two streams is immediately above the LF-1 arrow shown in Fig. 1. Both Fountain and Monument Creeks are perennial streams and sampling sites were established in the watershed by Herrmann et al. (2012); their locations indicated by global positioning system (GPS) and map descriptions are in Table 1. It is important to note that Fountain Creek is a major tributary to the lower Arkansas River in Colorado.

**Table 1:** Location of 14 sampling sites in the Fountain Creek watershed

Site	Lat (N)	Long (W)	Elevation (M)	Physical description of site location
UF-1	38.92691	−105.004	2334.77	UF at Green Mtn. Falls
UF-2	38.85948	−104.920	1940.66	UF at Manitou Springs, CO
UF-3	38.84629	−104.866	1859.89	UF at 26th St., Colorado Springs, CO
UF-4	38.83015	−104.842	1818.44	UF at 8th St., Colorado Springs, CO
MC-1	39.08271	−104.876	2097.33	MC at Mt. Herman Rd. near Monument, CO
MC-2	39.02415	−104.844	2031.80	MC at N. Entrance, U.S. Air Force Academy
MC-3	38.95424	−104.834	1942.80	MC at S. Entrance, U.S. Air Force Academy
MC-4	38.93322	−104.817	1924.20	MC at Woodman Road, Colorado Springs, CO
MC-5	38.84282	−104.828	1840.69	MC at Colorado College, Colorado Spring, CO
LF-1	38.81613	−104.822	1798.02	LF at Nevada St., Colorado Springs, CO
LF-2	38.60245	−104.670	1634.95	LF at S. Fountain, CO
LF-3	38.42975	−104.598	1532.84	LF at Pinon Bridge
LF-4	38.28793	−104.602	1432.86	LF at Hwy. 50, Pueblo, CO
LF-5	38.25572	−104.591	1414.88	LF upstream of confluence with Arkansas River, Pueblo, CO

*UF* Upper Fountain Creek, *MC* Monument Creek tributary and *LF* Lower Fountain Creek

### Collection of fish

Fish were collected using a Smith-Root LR-24 Electrofisher™ unit at each site with the section of stream sampled counter current: the distance varying by site until sufficient fish were captured. Fish were immediately removed from the net and euthanized using the cranial concussion (stunning) method as described in the 2007 American Veterinary Medical Association (AVMA) Guidelines. Each specimen was placed in a plastic bucket and carried to shore placed in a plastic bag then transferred to a cooler with wet ice for transport to the laboratory at CSU-Pueblo Aquatic Research Center (ARC). At the laboratory the fish were placed in a double zip-sealed plastic bag and frozen at −20 °C until they were thawed and processed for analysis.

We used the Colorado Parks and Wildlife designation to record each specimen collected. The scientific nomenclature, common names and the Colorado Parks and Wildlife designation were as follows: *Salmo trutta* (Brown Trout-LOC); *Semotilus atromaculatus* (Northern Creek Chub-CRC); *Catostomus catostomus* (Longnose Sucker-LGS); *Catostomus commersonii* (White Sucker-WHS); *Platygobio gracilis* (Flathead Chub-FHC); and *Campostoma anomalum* (Stoneroller-STR). One hundred fifteen fish, shown by their common names were collected in the Fountain Creek watershed and listed by the collection site (Table 2). Due to small sizes of some fish, only 111 were used in the analysis.

**Table 2:** One hundred fifteen fish, shown by their common names were collected in the Fountain Creek watershed

	Brown Trout	Northern creek chub	Longnose sucker	White sucker	Flathead chub	Stoneroller
UF-1	5					
UF-2	6					
UF-3	5					
UF-4	4		2			
MC-1		6		6		
MC-2		10	3	1		
MC-3		9	8			
MC-4		3	3			
MC-5				6		
LF-1	3		1	2		
LF-2			2	5		
LF-3			1	4		
LF-4				4	6	2
LF-5				4	4	
Totals	23	28	20	32	10	2

Because of the small sizes of some fish, only 111 were used for analysis of Se and Hg

### Collection and analysis of water samples

Prior to- and during the sampling of fish in 2009, 479 water samples were obtained at 14 sites in the watershed. The 2 years of sampling, 2007–2009 included 161 total (unfiltered), 205 dissolved (0.45 µM filter) and 113 pore (interstitial) water samples. The pore water samples were obtained using a mechanical vacuum-operated pore-water extractor similar to that described by Winger and Lasier (1991) and sampling method described by Herrmann et al. (2012). All samples were placed on ice, and transported to the laboratory where total and dissolved water samples were acidified with Ultrex (ultra-pure) concentrated HNO_3_ and stored at 4 °C until analysis. In the laboratory, the pore water was stored at 10 °C for 24 h to allow the particulates to settle; next clear supernatant was decanted into a clean 30 ml low density polyethylene sample tube acidified as above until analysis.

### Preparation of fish tissues and analysis

Fish were allowed to thaw at room temperature, removed from plastic bags and patted dry with clean paper towels. Each fish was placed on plastic wrap liners in acid-rinsed dissecting pan for inspection. Weights and lengths were recorded followed by a count of lateral line scales to differentiate between longnose (*C. catostomus*) and white suckers (*C. commersonii)*. After initial inspection, each fish was returned to the dissecting pan, an incision was made in the vent towards the head using stainless steel scissors to determine whether each fish was a gravid female, non-gravid female or male. From each of the 11 gravid females collected, ovaries, samples of skin, epaxial muscle and livers were removed and each positioned in individual weighing boats then placed in a Thelco model M drying cabinet at a temperature of 32 °C for 20 days. The wet to dry ratios of the 44 whole body homogenates averaged 0.267, a value close to the ratios of 0.284 for trout and 0.288 for non-game fishes reported by Fresquez and Ferenbaugh (1999). Therefore, all values of Se and Hg in the whole bodies of fish tissues are reported as wet weights by multiplying dry weights by a factor of 0.267. However, for the 44 individual tissues in the 11 gravid females, individual conversion factors were calculated for each ovary, skin, epaxial muscle and liver tissue. As an example of a conversion, the liver of the single gravid brown trout had a dry to wet conversion factor of 0.252.

The whole-bodies of non-gravid fish were cut into smaller sections, placed in an Osterizer Galaxie™ blender, equipped with a stainless steel blade, plastic jar and plastic lid, and homogenized. Before any fish were processed, detergent and water were placed in the blender and operated for approximately 30 s. After the unit was dissembled, each part was washed again with detergent and tap water then rinsed with hot tap water followed by a rinse with 18.2 MΩ deionized water from a Millipore Simplicity™ water polisher hereafter referred to as deionized water. Next, all parts of the blender were acid rinsed using a solution prepared by adding 5 ml conc. HNO_3_ plus 20 ml conc. HCl diluted to 1 l with deionized water. Next, each part of the blender was rinsed twice with deionized water; and after the blender was assembled, rinsed twice again, with deionized water. Finally, to ensure no cross contamination, a “blender” blank was prepared and analyzed for Se and Hg contamination. Our “cleaning procedure” showed no evidence of Se or Hg contamination between the preparations of each fish for analysis. Finally, all dissecting tools used in the procedure were washed with detergent and water, rinsed with the HNO_3_:HCL solution, and rinsed with deionized water as described above.

In some cases, small fish specimens were processed more easily by adding an additional ml of de-ionized water to 1 g of fish wet weight. Once the homogenate of each fish consisting of pieces >1 mm, a Teflon stirring tool was used to remove a portion of the tissue-slurry into a weighing boat. Tissue boats were placed in the drying oven at 32 °C for 30 days. There was sufficient (wet) tissue-slurry to provide approximately 1 g of dry tissue for further analysis.

Analysis of tissue- and water samples was as follows. After drying approximately 100 mg of dried each tissue was transferred into a Teflon microcentrifuge tube reaction vessel and digested according to U.S. EPA (2008) protocol (U.S. EPA document 846-SW) using a Milestone Ethos EX™ microwave. Digestion consisted of weighing approximately 100 mg of dried tissue into a clean Teflon microwave digestion tube. One ml of concentrated HNO_3_, 1.0 ml of 35 % H_2_O_2_ and 100 µl concentrated HCL was added to each reaction vessel, then sealed and placed inside a microwave insert. Each insert and vessel content was heated to 180 °C for 30 min and held another 10 min for cooling. Digested samples were transferred to Falcon tubes, and the volume adjusted to 10 ml. Samples were then diluted 1:10 and analyzed for Hg and Se using an Agilent model 7500CE™ inductively plasma mass spectrometer (ICP-MS). Initially, National Bureau of Standards bovine liver (SRM 1577a) was used as the standard vertebrate tissue to determine digestion efficiency and recovery of total Hg and Se. In addition, we evaluated the accuracy of our analytical methods for Hg and Se by including two certified reference materials: DOLT-5 (dogfish liver from National Research Council Canada, Ottawa Canada) certified as 0.44 ± 0.18 µg/g Hg dry weight and 3.45 ± 0.40 µg/g Se dry weight and DORM-4 (fish protein from National Research Council Canada, Ottawa, Canada) certified as 0.412 ± 0.036 µg/g Hg dry weight and 3.4 ± 0.40 µg/g Se dry weight. For the fish tissues analyzed, known additions were used for each batch of 50 samples. The percent recovery of the Hg from the DOLT-5 reference was 95.4 % and from the DORM-4 reference, 90.2 %. The percent recoveries of Se from the two reference materials were 100 % from DOLT-5 and 110/4 % for DORM-4. The detection limits for fish tissue were 6.81 parts-per-trillion (ng/kg, ppt) for Se 78 and 1.37 parts-per-trillion (ng/kg, ppt) as Hg 202. For this report, Se and Hg in fish tissues will be reported as wet weights (ww). For the water samples, multi-element environmental external calibration standards as well as internal standards, were purchased from Inorganicventures ™ and diluted with 1 % nitric and 0.5 % hydrochloric acid prior to analysis. There were 15 field blanks and eight laboratory blanks analyzed during the analysis of the water samples, and the known additions method was used for determining the detection limits. For water samples, the detection limits were 3.18 (ng/l, ppt) for Se-78 and 3.29 (ng/l, ppt) for Hg-202. In this report we will report the results as total mercury or Hg recognizing that both the elemental and methylated forms of the metal are included.

### Analysis of data

As stated earlier, this study was a matter of reconnaissance and discovery related to the question of whether Se would be found in the whole bodies of fish throughout the Fountain Creek watershed and if so, to what extent was the accumulation? (However, not anticipated was finding mercury in all fish). Our initial plan was to collect, as much as possible, a single fish species from 14 sites for comparisons of contamination by site, or at least, comparison of stream segments; however, collecting a single species was not possible because upper Fountain and Monument Creeks begin as cold water streams with markedly different assemblages of fish. Therefore, this report will focus on baseline data for selenium and mercury in the tissues of brown trout, a cold-water species and five warm-water species plus an initial study on the localization of Se and Hg in four selected tissues including the ovaries. We were not able to subject the data to rigorous statistical analysis but we were able to indicate trends of tissue-Se and tissue-Hg in fish whole bodies and selected tissues. We used regression analysis to determine whether a direct or inverse relationship existed between concentrations of Se and Hg in the whole bodies of fish at the 14 sites.

## Results and discussion

Six species of fish totaling 115 specimens were collected and are listed by their common names and collecting sites in the watershed (Table 2). Because of small sizes in four specimens, only 111 fish were used for analysis of Se (and concurrently, the Hg). In addition, we found 11 of the 111 fish representing six species with mature ovaries, collected throughout the watershed, that could be used for a comparisons of Se (and Hg) in other tissues such as the skin, muscle and liver. Condition factors (an indicator of healthy fish in the watershed) were all >1.0 suggesting all species were healthy in the watershed and are listed as follows: Brown Trout 1.17 (SD 0.30); Northern Creek Chub 1.22 (SD 0.14); Flathead Chub 1.01 (SD 0.09); Long nose Sucker 1.01 (SD 0.11); Stoneroller 1.08 (SD 0.18); and White Sucker 1.12 (SD 0.10).

**Fig. 2:**
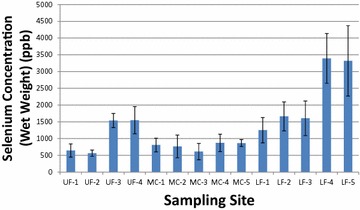
Average selenium (µg/kg, ppb) are represented by the blue vertical bars and brackets, one standard deviation in whole-bodies of fish from the Fountain Creek watershed, Colorado, collected in March through May, 2009. Twenty brown trout and two longnose suckers were collected at sites UF-1-through UF-4, a cold-water segment of Fountain Creek; however, three brown trout as well as several warm-water fish were collected below the confluence of Upper Fountain Creek and Monument Creeks at site LF-1. All other (warm-water) fish were collected in the warm-water segments of Monument and Lower Fountain Creeks

**Fig. 3:**
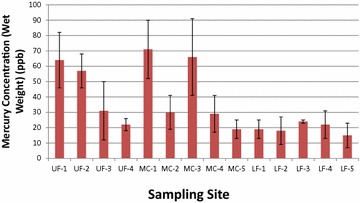
Average concentration of mercury (µg/kg, ppb) are represented by the *red vertical bars* and *brackets* represent one standard deviation in whole-bodies of fish from the Fountain Creek watershed, Colorado, collected in March through May, 2009. Twenty brown trout and two longnose suckers were collected in sites UF-1-through UF-4, a cold-water segment of Fountain Creek; however, three brown trout as well as several warm-water fish were collected below the confluence of Upper Fountain Creek and Monument Creeks at site LF-1. All other fish were collected in the warm-water segments of Monument and Lower Fountain Creeks

**Fig. 4:**
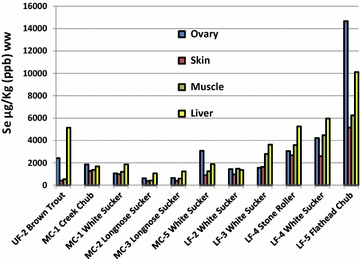
Concentrations of selenium (µg/kg, ppb, ww) in ovarian, skin, muscle and livers of individual fish from 11 sites are shown. Noteworthy at site MC-1 there are data for a creek chub and white sucker and at site LF-4, data from a stone roller and a white sucker; all other data shown by the *bars* were single fish at each of the other sites. We did not find gravid fish at all sites

**Fig. 5:**
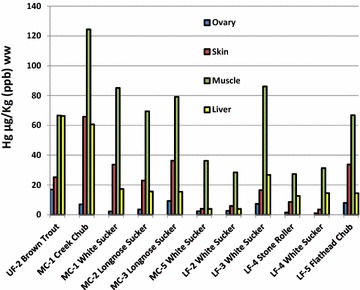
Concentrations of mercury (µg/kg, ppb, ww) in ovarian, skin, muscle and livers of individual fish from 11 sites are shown. Note that at site MC-1 there are data from a creek chub and white sucker and at site LF-4, data from a stone roller and a white sucker; all other data shown by the bars were single fish at each of the sites. We did not find gravid fish at all sites

We arranged the data in the following figures to reflect the orientation of the watershed which is situated generally north to south and three distinct segments. Because the basin is in the form of a “Y” we arranged the Se and Hg data in Figs. 2, 3, 4, 5 to reflect the distinctive nature of the watershed. The Upper Fountain Creek data (UF) are arranged to the left; the Monument Creek data (MC) are in the middle; and the Lower Fountain Creek data, representing the combined flows of the two segments, are shown as the lower (LF) series of sites. The Se concentration (µg/kg) in the whole bodies of fish at UF-4 and MC-5 appear to be similar to those after the confluence at LF-1 (Fig. 2). From site LF-1 downstream, Se in the fish showed a marked increase in the metalloid at all the LF-sites, especially at the LF-4 and LF-5 sites located in the City of Pueblo, Colorado. Whole body concentrations of mercury (µg/kg) are shown in Fig. 3. Generally, mercury was higher in fish collected from the upper segments of Fountain Creek and Monument Creeks; and note that the flow-volumes at sites UF-4 and MC-5 are joined immediately upstream of site LF-1. Also note that the average Hg concentrations in the whole bodies of fish at the confluence of the two stream segments appear similar to those downstream of the confluence. The highest whole-body selenium was measured in northern creek chubs and white suckers in Pueblo, Colorado at site LF-4 (3393 µg/kg, dry weight; 906 µg/kg wet weight; Fig. 2; Table 2); the highest mercury was in white suckers, flathead chubs and stonerollers from the Monument Creek tributary north of Colorado Springs Colorado at MC-1 (71 µg/kg, dry weight; 19 µg/kg wet weight; Fig. 3; Table 2).

Concentrations in the whole bodies of Se (Fig. 2) appeared to increase with stream distance downstream and Hg (Fig. 3) tended to decrease which poses the question whether there was a significant inverse relationship between the two. However, in this study, regression analysis did not show a significant interaction (positive or negative) between the mercury and selenium in the whole bodies of the fish in the Fountain Creek watershed. A review of Se and Hg interactions by others suggested that neither positive nor negative correlations are consistent (Stewart et al. 2010). In the Stewart et al. (2010) review, three investigators found no correlation between Se and Hg while two investigators found a negative correlation. In order to find correlations between tissue-Se and tissue-Hg, our survey probably had insufficient numbers of specimens, differing species compositions collected at sites, and no single year-class comparisons. Furthermore, the three segments of the watershed had differing elevations, water temperatures (cold, cool and warm-waters) and food bases.

Eleven female fish with mature ovaries along with samples of skin, muscle, and liver are shown in Fig. 4 for Se. Selenium was highest in the livers of the fish at eight sites whereas the element was highest in the ovaries of fish at three sites. However, in the fish where the ovaries were the highest, the livers were always second highest. For example, at MC-1, the upper site in Monument Creek, Se in the liver was 1854 µg/kg in the ovary compared to 1680 µg/kg in the liver. In the white sucker at LF-2 there was 1435 µg/kg in the ovary compared to 1358 µg/kg in the liver, and the flathead chub at LF-5, there was 14,672 µg/kg in the ovary but 10,672 µg/kg in the liver. In summary, it may be an important discovery in the Fountain Creek watershed to find a pronounced localization of Se in the livers of the 11 fish rather than finding the metalloid in the ovaries. Despite not being able to capture the same species at all sites in the watershed necessary for direct comparisons, Se tended to decrease in the four tissues in Monument Creek sites (MC-1 to MC-3) but tended to increase in MC-5 and lower Fountain Creek sites (LF-2 through LF-5).

There is circumstantial evidence that Se in lower Fountain Creek (Fig. 1) could be affecting populations of fish based on the comparison of Se criteria (concentrations) in fish tissues and ambient water samples U.S. EPA (2015). The criteria document proposes national ambient water quality criteria as follows: (1) the concentration of Se in the eggs or ovaries of fish does not exceed 15.8 mg/kg, dry weight (dw); (2) the concentration of selenium (a) in whole-body of fish does not exceed 8.0 mg/kg dw, or (b) in muscle of fish (skinless, boneless fillet) does not exceed 11.3 mg/kg dw; (3) the 30-day average concentration of selenium in water does not exceed 3.1 µg/l in lotic (flowing) waters and 1.2 µg/l in lentic (standing waters more than once in 3 years on average); and finally, (4) an intermittent exposure of Se in the water column of the element lentic or lotic more than once in 3 years on average.

Therefore, listed below are the comparisons for Se in ovaries, whole-body tissues, muscle and in water samples from Fountain Creek that can be compared to the draft national criteria above.

- Se was close to the ovarian criterion in the white sucker at MC-5 (11.5 mg/kg dw) or 3.07 mg/kg [wet weight (ww)]; it exceeded the ovarian limit in the white sucker at LF-4 (15.79 mg/kg dw or 4.22 mg/kg ww); and Se exceeded the ovarian limit in the flathead chub at LF-5 (55 mg/kg dw or 14.67 mg/kg ww) (See Figs. 1, 4).
- Whole bodies of fish at LF-3 were (6.02 mg/kg ww or 1.61 mg/kg ww); whole bodies of fish at LF-4 were (12.71 mg/kg dw or 3.39 mg/kg ww); whole bodies of fish at LF-5 were 12.44 mg/kg dw or 3.30 mg/KG ww (see Fig. 2).
- Se was close to the muscle criterion in the white sucker at LF-3 (10.46 mg/kg dw or 2.79 mg/kg ww); it was close to the muscle criterion in the stone roller at LF-4 (10.87 mg/kg dw or 2.0 mg/kg ww); but it exceeded the muscle criterion in the white sucker at LF-4 (16.73 mg/kg dw or 4.47 mg/kg ww); and it exceeded the muscle criterion in the flat head chub at LF-5 (23.39 mg/kg dw or 6.25 mg/kg ww) (see Fig. 4).
- Some samples of water (pore, dissolved and total Se) exceeded the 3.1 µg/l criterion limit at site MC-5, LF-2, LF-3, LF-4, and LF-5. At sites LF-4 and LF-5, both within the city of Pueblo, Colorado, all samples were above the 3.1 µg/l criterion limit (see Fig. 1; Table 3).

**Table 3:** Ranges of selenium (µg/l) in water samples at each site and the number (#) of samples at that site

Site	# Pore^a^ and range	# Dissolved^b^ and range	# Total and range
UF-1	8 0.03–0.25	14 BDL–0.24	13 BDL–0.25
UF-2	6 0.09–0.49	13 BDL–0.19	12 BDL–0.18
UF-3	18 BDL–0.93	19 BDL–0.95	17 BDL–0.98
UF-4	19 BDL–1.41	20 BDL–1.89	16 BDL–1.87
MC-1	6 0.18–1.43	14 BDL–0.26	10 0.11–0.27
MC-2	6 0.15–0.29	17 BDL–0.17	16 0.14–0.37
MC-3	6 0.33–2.38	17 BDL–0.67	14 0.29–0.55
MC-4	7 0.11–2.72	13 BDL–0.89	9 0.40–0.85
MC-5	6 2.38–4.45	13 0.31–5.68	8 1.51–5.01
LF-1	7 5.89–8.70	13 BDL–4.42	9 1.54–4.52
LF-2	6 3.06–3.27	12 BDL–4.52	9 2.67–4.42
LF-3	6 2.93–3.32	13 BDL–5.59	9 2.97–5.71
LF-4	6 16.11–21.55	14 8.89–20.25	10 8.46–19.16
LF-5	6 8.14–11.85	13 6.48–17.55	9 7.60–21.12

The detection limit was 3.18 ng/l and values below that limit are indicated as BDL

^a^Often referred to as interstitial water samples

^b^Dissolved samples of water were filtered through a 0.45 µM filter; total-water samples were unfiltered. Selenium was detected in 416 of the 479 (87 %) in the total, pore and dissolved water samples collected between 2007 and 2009

Concentrations of mercury are shown in fish tissues (as above with Se) are shown in Fig. 5. With respect to Hg in the four tissues of the female fish, we found it remarkable that muscle was the highest in all cases; the exception being the brown trout where the liver was virtually equal to the muscle at the UF-2 site; 66.6 µg/kg in the muscle versus 66.3 µg/kg in the liver (Fig. 5). None of the other three tissues had Hg concentrations approaching those in the muscle of the other warm-water fish, however, the flathead chub at site LF-5 immediately above the confluence with the Arkansas River in Pueblo had about half that in the muscle (33.7 µg/kg). Interestingly, the average concentration of Hg in the muscle did not seem to vary greatly despite the diversity of species and locations where they were collected (six species collected from nine sites). The average concentration of Hg in the muscle of all fish was 63.7 (SD 30.5 µg/kg).

We compared the whole body concentrations of Hg in fish collected from the 14 sites in Fountain Creek (shown in Fig. 3) to the “threshold-effect tissue concentrations” of 200 µg/kg (wet weight) established by Sandheinrich et al. (2011). The benchmark was established from more than 43,000 measurement of fish from 2000 locations in the Great Lakes region. We noted that the average concentration of Hg in fish from Fountain Creek were well below the established benchmark (Fig. 3); however, some individual fish approached about half of the benchmark value, particularly at sites MC-1 and MC-3. It should be noted that the fish at UF-1 and UF-2 were brown trout with whole-body Hg concentrations approaching 1/3 of the benchmark concentration.

Based on the difference in concentrations of Se versus Hg in the watershed, it was not surprising that Se was more available to biota (Tables 3, 4). Of the 479 water samples collected from the Fountain Creek watershed from 2007 to 2009, 416 had Se above the detection limit of 3.18 ng/l. By comparison, mercury was detected in only 20 of the 479 water samples (detection limit of 0.001 ng/l). Additionally, selenium was detected in total, dissolved and pore water samples at all 14 sites whereas Hg was found only in samples from the lower Fountain Creek (LF-2–LF-5 segment sites). Furthermore, Hg was found in the total and dissolved samples and not in the pore water samples; probably due to frequent scouring along the steep slopes in the upper reaches of the streams but also due to constant shifting of the sandy, cobble and gravel substrates when the watershed became a plains stream.

**Table 4:** Ranges of total mercury (µg/l) in water samples at each site and the number (#) of samples at that site

Site	# Pore^a^ and range	# Dissolved^b,c^ and range	# Total^b,c^ and range
LF-2	6 BDL	12 BDL–0.03	9 BDL–0.02
LF-3	6 BDL	13 BDL–0.01	9 BDL
LF-4	6 BDL	14 BDL	10 BDL–0.02
LF-5	6 BDL	13 BDL	9 BDL–0.01

The detection limits were 3.29 ng/l and values below that limit are indicated as BDL

^a^Often referred to as interstitial water samples

^b^Mercury was only detected in 20 (4 %) of the 479 total, pore and dissolved water samples collected between 2007 and 2009. Mercury was not detected in any of the pore-water samples and the BDL’s are shown to compare to the concentrations of mercury in the dissolved and total samples. Mercury was not detected at LF-1 but was detected at site LF-2 which is below the Colorado Springs, Colorado Las Vegas waste-water treatment plant. Sites LF-2 and LF-3 are in an agricultural setting, land usage primarily irrigated crops and grazing. LF-4 and LF-5 are within the City of Pueblo, Colorado

^c^Dissolved samples of water were filtered through a 0.45 µM filter and total-water samples were unfiltered

Various geological descriptions and studies of the Fountain Creek watershed suggested Se could be an important environmental factor affecting resident biota in Fountain Creek. Presser et al. (1994) noted the potential for ecological damage of selenium in six states, one of which was at the Colorado/Kansas border; the location on their map was identified as the Middle Arkansas River Basin. Chabrillat et al. (2002) identified and mapped the expansive clay soils of the Front Range Corridor in Colorado including the Fountain Creek Basin and also showed the proximity of the bedrock formations, abundance of montmorillonite (clay) and Pierre shales surrounding the Fountain Creek Basin (Fig. 1 shows the colors of bedrock shale and shale mixed with bentonite). Kulp and Pratt (2004) characterized the Pierre shale by its “anomalously high concentrations” of naturally occurring Se. Bossong (2001) sampled Fountain and Monument Creeks between 1987 and 1997 and reported 49 cases of in-stream exceedences of >5.0 µg/l as total Se; all samples were obtained between Colorado Springs and Pueblo, Colorado. Van Derveer and Canton (1997) reported Se in water August and November of 1994 of 7.0 and 6.0 µg/l at Pinon (LF-3) and also reported 18 and 7.0 µg/l in Pueblo, Colorado. Divine and Gates (2006) reported total and dissolved Se concentrations ranging from 1.3 to 64.4 µg/l; the mean and median values of 5.4 and 3.6 µg/l respectively. Similarly, Turner (2009) found dissolved Se in water from site LF-4 (Lower Fountain Creek, Pueblo, CO, Table 1) averaging 9.7 µg/l in spring and 18.6 µg/l in the fall. As part of an extensive monitoring study Herrmann et al. (2012) found bioconcentration factors in the aquatic plant, *H. ochraceum*, exposed for 10 days in-stream, as high as 5.8 × 10^3^ at Green Mountain Falls site (UF-1) and 1.5 × 10^4^ at the Manitou Springs site (UF-2) in the fall of 2007. The main conclusion by Herrmann et al. (2012) was that the aquatic plants (*H. ochraceum*) used in the study were suitable indicators of Se-bioavailabilty and that Se potentially would enter additional trophic levels, including fish, in Fountain Creek. Therefore, finding Se in six species of fish at all 14 sites in Fountain Creek was not surprising.

However, mercury in the fish was not anticipated and the following are some possibilities as to sources of the metal. Mercury could be in treated waste water from multiple municipal treatment facilities in the upper basin; second, Hg could have come from natural weathering of upper basin minerals. A third possibility could be erosion from historical mining activities around Colorado Springs and the fourth could be the atmospheric deposition of Hg, emitted during the generation of power in Colorado Springs and downstream at Pueblo.

In the review of the literature, we found only two reports of Hg being found in surface waters of Fountain Creek. The first was by Chafin (1996) who questioned whether the surface-water quality affects the recharge entering the alluvial aquifer and also noted that Fountain Creek receives “sewage effluent” near the northern boundary selected for study. Although Chafin (1996) reported few samples above the 0.1 µg/l detection limit during the August of 1988 and February of 1989 collections, some were above the detection limit. All samples were below the detection limit at the Nevada Street site in Colorado Springs (our LF-1 site) immediately below the confluence of Upper Fountain Creek and Monument Creek (see Fig. 1; Table 1 for description and location); however, concentrations of Hg were 0.2 µg/l at sites SW2 and SW3 on August 17, 1988; both approximately eight kilometers above our LF-2 site (see Fig. 1; Table 1). In our samples, we also detected Hg in this stretch of Fountain Creek but at much lower concentrations; values of 0.03 ng/l in the dissolved fraction and 0.02 ng/l in the total fraction in surface water samples. Furthermore, Chafin (1996) on February 1, 1989 reported 0.2 µg/l at the Pinon Bridge (his SW5 site; the same site as our LF-3 site (see Fig. 1; Table 1 for description and location). In comparison, at the Pinon site (LF-3) we found 0.01 ng/l Hg. In both Chafin’s (1996) study and our study, Hg was only detected in surface waters from Fountain Creek below the City of Fountain, Colorado. A second investigator, Bossong (2001), between October 1987 and September 1992 reported five instances where mercury exceeded the 0.1 µg/l standard in the dissolved fractions below all of the major treatment plant discharges. Bossong’s (2001) stations were identical to our sites at LF-1, LF-3, and LF-5 (see Fig. 1; Table 1 for descriptions). At the LF-1 site, Bossong (2001) reported 0.50 µg/l Hg; at LF-3 he reported 0.20 µg/l; and at LF-5 he reported 0.50 µg/l. In addition, on two occasions below the Colorado Springs Las Vegas treatment plant contribution, several kilometers above our LF-2 site, Bossong (2001) reported 0.20 and 0.30 µg/l Hg. Therefore, at the five sites (LF-1-LF-5) constituting the lower Fountain Creek, both Chafin (1996) and Bossong (2001) in earlier studies, corroborate our finding Hg above the instrumental detection limit at four of the same sites. We find it incongruent for three investigations to report Hg at detectable concentrations only in the lower segment of the Fountain Creek segment, but regarding Hg in the fish tissues, in this reconnaissance, we found Hg in fish considerable greater in the upper reaches of the watershed (Fig. 3).

Concerning Hg from natural sources, a review did not mention the use of mercury in the mining activity or the presence of the mineral, cinnabar (HgS) in the Fountain Creek Basin. Nevertheless, an assayer (a Mr. Brown) in the heavily-mined area of Cripple Creek, Colorado to the west of Colorado Springs, reported a “little cinnabar, with native mercury” in the Moon Anchor vein. Brown noted at the time that, “this is the only reported occurrence of this mineral in the district” (Lindgren and Ransome 1906). The City of Cripple Creek and surrounding mines are located near the upper reaches of Fountain Creek but they are not in the Fountain Creek watershed. The mining was actually in the Fourmile Creek Watershed sloping to the south, and runoff in that basin eventually confluences with the Arkansas River in Canon City, Colorado.

We questioned whether wind and water erosion from a historic gold-processing operation was responsible for some elevated mercury in Fountain Creek. Mercury was reported in Fountain Creek above our study site, UF-4 by the U.S. EPA (1994) in a report about the Gold Hill Tailings Site, known locally as Gold Hill. The tailings mound was formed from ore shipped from the Cripple Creek area of Colorado, then “roasted” at Old Colorado City, now a historic section west of downtown Colorado Springs. According to the U.S. EPA (1994), ore from Cripple Creek was processed using combinations of bromide, roasting, cyanide and flotation for ore containing gold. During its 45-years of operation (about 1904–1949), gold was extracted from 14.5 million tons of ore then mounded around the site. According to U.S. EPA (1994), soil samples from the northeast face of the tailings pile (that slopes down to become a stream bank of Fountain Creek) contained cyanide and numerous heavy metals, including elevated cyanide, arsenic, copper, lead, silver and mercury compared to offsite background soil samples. During our sampling of Fountain Creek above and below Gold Hill dust from the hill was being carried by winds either upslope in the Fountain Creek Basin as well as down slope into the valley below where Monument Creek confluences with upper Fountain Creek. It seems reasonable that both wind- and water-borne mercury could enter basin water and eventually move into resident biota. Of five of 22 samples of soil samples, two at stream-side of Fountain Creek (samples, S-4 and S-5) had mercury at detectable concentrations (Dames and Moore 1999). But it is important to note that we did not detect Hg in any water samples collected from the Upper Fountain Creek sites neither downstream nor upstream of Gold Hill. However, we found Hg to be present in the whole bodies of fish collected from sites UF-4, MC-5 and LF-1, closest to Gold Hill, but the concentrations were relatively low compared to sites UF-1, UF-2, MC-1, MD-3 upstream in Fountain and Monument Creeks (see Fig. 3 for comparison of data and Fig. 1 for locations).

**Fig. 6:**
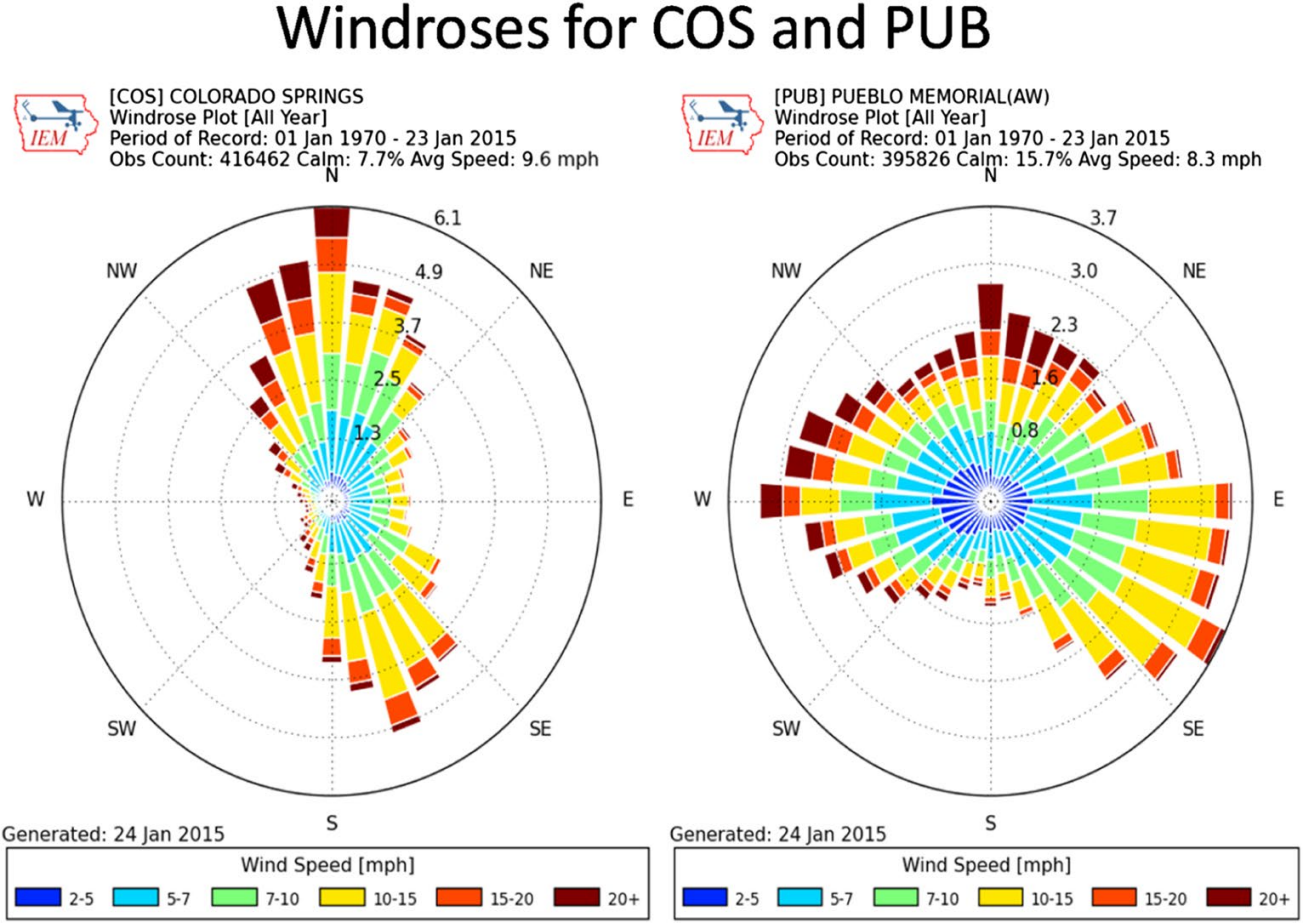
**a** COS and **b** PUB. *Wind roses* indicate the dominant wind directions and the direction of strongest wind speeds. These plots were recorded at the Colorado Springs, Colorado municipal airport (COS) and the Pueblo, Colorado municipal airport (PUB). The period of record is from January 1, 1970 to January 23, 2015

Regarding the atmospheric deposition of Hg from power plants in a watershed, the hypothesis of an “airshed” might be useful to consider in this discussion. Driscoll et al. (2006) provided several definitions of an airshed admitting that the concept is not easily understood because (1) it is three-dimensional and (2) possesses a time-variant temperament of atmospheric flow. The authors suggested that “one definition of an airshed is based on assumptions about wind flow patterns surrounding a location of interest and the length of time that a substance is transported in the atmosphere”. True, it is probably easier to understand a watershed rather than an airshed because a contaminant entering or moving through- or into an airshed could appear insignificant to a casual observer. But by considering the movement of air over an airshed for decades of weather conditions, most might eventually understand that any contaminant carried by air during an extended time possibly will be significant. For example, 44 years of data, collected from Colorado Springs and Pueblo, Colorado available from the Colorado Climate Center (Newman 2015) showed prevailing winds could have carried mercury-laden aerosols from three coal-fired power generating plants and the Gold Hill site discussed above (Fig. 6a, b). Wind rose plots provided average frequencies of wind directions and speeds for Colorado Springs and Pueblo that might indicate dispersion of Hg onto soils and sediments of the Fountain Creek watershed or if you will, the airshed. Over time, through microbial action, the methylation of mercury (MeHg) and bioaccumulation by aquatic biota could occur at the top of the food chain (Weiner et al. 2003). Further, during the operation of 45 years of gold extraction at the Gold Hill site discussed above, there could have been air-borne aerosols carried upslope from the roasting stack, and Hg deposited along the upper slopes in the upper Fountain Creek (Ute Pass) valley. There is a recent precedent for considering Hg transport by directional winds because Hissler and Probst (2006) reported mercury uptake in bryophyte-plants used in their study was a function of distance from a chlor-alkali industrial site in France.

An inspection of the wind roses showed the prevailing wind direction in Colorado Springs was from the north; however, a substantial portion of the time, it was from the south–southeast. Mercury concentrations in the fish taken from the Upper Fountain Creek (UF-1 through UF-4) could have been due to aerosols carried by the southeasterly winds from the Martin-Drake power plant located in downtown Colorado Springs. Southeasterly winds could have also carried the Hg northward along Monument Creek (see MC sites and Fig. 2). Nevertheless, the stronger prevailing northerly winds may have borne the Hg southward and deposited Hg along the Fountain Creek segment sites (see LF-1 through LF-3, Figs. 1 and 6b) towards the city of Pueblo, Colorado. In addition, Hg may be carried by prevailing winds into the Fountain Creek Basin South of Colorado Springs from the Ray Nixon coal-fired generating plant, located approximately 24 km south of the Martin-Drake plant.

In contrast to north and southeast directional winds in Colorado Springs, the wind rose summary in Fig. 6b shows prevailing winds in Pueblo have generally been from the east–southeast during the eight warmest months of the year; prevailing winds were from the west during the four winter months. Therefore, mercury-aerosols from the Comanche coal-fired power generating plant in Pueblo could have been deposited at sites LF-3 through LF-5 in lower Fountain Creek, Pueblo, Colorado. There is a precedent for finding Hg dispersal over large areas because Berg et al. (1995) demonstrated the utility of mosses as “collectors” of metals after measuring 33 elements (including mercury) from atmospheric deposition in southern Norway.

Brigham et al. (2009) indicated that atmospheric deposition was the source of mercury along streams, and characteristics of the streams basins appeared as important factors for transforming total (elemental) Hg into the methylated form (MeHg) thereby making it more available to aquatic food bases. Further, the research team showed that streams with greater drainage basins along with more wetlands had the highest pore water dissolved organic carbon; the latter an indicator where methylation of mercury is enhanced (Marvin-Di Pasquale et al. (2009). Their results also showed that Hg-contamination at higher trophic levels of fish in streams was likely dominated by the MeHg available at the base of the food chain (or web) rather that the trophic position of the predator fish species (Chasar et al. 2009). In the Fountain Creek watershed, it is possible that in upper reaches of the two streams that confluence (upper Fountain and Monument Creek), could favor more methylation of total mercury by microbial activity in the wetlands and marshes of the tributaries in the upper basin rather than in the lower. There are about 18 intermittent or permanent tributaries emptying into upper Fountain Creek basin and about 20 tributaries in Monument Creek. By contrast, lower Fountain Creek is mainly a “shifting, shallow sandy-bottom plains-stream” with scattered wetlands and marshes present only in still-water oxbows, scoured by previous high-water and flood conditions. The biological contents of the wetlands only enter the main stream flowages during the next flood event. Clearly, we cannot identify point- and nonpoint-sources of Hg entering the relatively small Fountain Creek watershed in Colorado, but at this juncture, we agree with Peterson et al. (2007) that it appears likely much of the Hg in fish is from atmospheric deposition both local and sources to the west. We have noted that Obrist et al. (2008) have affirmed the following, “not surprisingly, contributions are modeled to be highest in the western U.S. with Asian sources accounting for 27 % of measured mercury depositions in Colorado, for example.”

## Conclusions

This reconnaissance of selenium and the unanticipated finding of mercury suggest future monitoring effort in the Fountain Creek Basin should consider the following questions: (1) could trends of increasing Se in the whole bodies and tissues of fish in the watershed be inversely associated with decreasing Hg if samples of fish were increased, (2) is it possible that the ratio of high Se in eight ovaries of some fish compared to the high Se in the livers of other fish hold true if, again, the number of fish-sampled were increased; further, could this ratio vary among the species collected, and (3) should the Se and Hg localization in more than four tissues of fish (including stomach contents) be investigated? Perhaps the most intriguing question from this reconnaissance was (4) explaining the mechanism leading to exceptionally high concentrations of Hg in the whole-bodies of fish in the upper reaches of both Fountain and Monument Creeks and yet, Hg was only detected (occasionally) in samples of water obtained from the lower stretch of the watershed.

Nevertheless, finding Se and Hg in all whole bodies and the four tissues of the fish showed addition monitoring of aquatic biota should commence in the Fountain Creek Basin. Recent monitoring of brown trout in the upper segment of Fountain Creek showed both elements in 11 tissues as well as the stomach contents strongly indicated that diet and not ambient water was the major source of Hg and Se bioaccumulated by the fish (Herrmann et al. 2015). Furthermore, average selenium in fish livers were higher than ovarian tissue at the uppermost site and at two lower sites in the watershed suggesting that liver tissue, in addition to egg-ovary, should be utilized as an indicator tissue for Se toxicity (Herrmann et al. 2015). We suggest that research commence to identify and quantify Hg from natural sources (if any are discovered) compared to the anthropogenic sources. To accomplish this task, as many components of the watershed as possible should be included in the study by sampling organisms from various trophic levels such as plants and macroinvertebrates. Further, the use of isotopic analysis, using ICP-MS and Multi-collector ICP-MS technology could enable investigators to “fingerprint” the isotopes of Hg in plant and animal tissues as to their origins in the watershed.
